# Temperature-Independent Current Dispersion in 0.15 μm AlGaN/GaN HEMTs for 5G Applications

**DOI:** 10.3390/mi13122244

**Published:** 2022-12-17

**Authors:** Nicolò Zagni, Giovanni Verzellesi, Alessandro Chini

**Affiliations:** 1 Department of Engineering “Enzo Ferrari”, University of Modena and Reggio Emilia, via P. Vivarelli 10, 41125 Modena, Italy; 2 Department of Sciences and Methods for Engineering (DISMI) and EN&TECH Center, University of Modena and Reggio Emilia, via G. Amendola, 2, 42122 Reggio Emilia, Italy

**Keywords:** GaN HEMTs, 5G, current collapse, T-independent process, Fe doping, TCAD simulations

## Abstract

Thanks to high-current densities and cutoff frequencies, short-channel length AlGaN/GaN HEMTs are a promising technology solution for implementing RF power amplifiers in 5G front-end modules. These devices, however, might suffer from current collapse due to trapping effects, leading to compressed output power. Here, we investigate the trap dynamic response in 0.15 μm GaN HEMTs by means of pulsed I-V characterization and drain current transients (DCTs). Pulsed I-V curves reveal an almost absent gate-lag but significant current collapse when pulsing both gate and drain voltages. The thermally activated Arrhenius process (with *E*_A_ ≈ 0.55 eV) observed during DCT measurements after a short trap-filling pulse (i.e., 1 μs) indicates that current collapse is induced by deep trap states associated with iron (Fe) doping present in the buffer. Interestingly, analogous DCT characterization carried out after a long trap-filling pulse (i.e., 100 s) revealed yet another process with time constants of about 1–2 s and which was approximately independent of temperature. We reproduced the experimentally observed results with two-dimensional device simulations by modeling the *T*-independent process as the charging of the interface between the passivation and the AlGaN barrier following electron injection from the gate.

## 1. Introduction

Short-channel length AlGaN/GaN high-electron mobility transistors (HEMTs) are an interesting technology option for realizing highly efficient power amplifiers in 5G communications systems, due to the high power/frequency handling capability and efficiency they offer [1]. Operation at high-voltage levels might induce the so-called “current collapse” effect that compresses the RF output power—thus limiting efficiency—and can trigger trapping effects in different regions of these devices [2,3,4,5,6]. Careful analysis of the physical mechanisms inducing such detrimental power compression is therefore required.

Here, we investigate the current-collapse effects in 0.15-μm AlGaN/GaN HEMTs by means of electrical characterization in terms of pulsed I-V curves and drain current transients (DCTs). Concerning the pulsed characterization, comparison between pulsing from the (*V*_GS,B_,*V*_DS,B_) = (0, 0) V baseline and from (*V*_GS,B_,*V*_DS,B_) = (*V*_TH_–1, 0), (*V*_TH_–1, 28), and (*V*_TH_–1, 56) V (*V*_TH_ is device threshold voltage). Remarkably, the devices-under-test (DUTs) exhibited negligible gate-lag [2], while showing a significant current collapse when pulsing both gate and drain voltage from OFF to ON state. To obtain information about the involved traps, we performed DCT characterization [7,8] at different temperatures by applying short (1 ms) and long (100 s) trap-filling pulses in the OFF-state (*V*_GS,OFF_, *V*_DS,OFF_) = (*V*_TH_–1, 56) V and monitor drain current transient response during recovery at quiescent point conditions (*V*_GS,Q_, *V*_DS,Q_) = (−1, 2) V for up to 100 s. After applying the short trap-filling pulse, the typical DCT due to electron emission from traps associated with Fe-doping in the buffer was observed [9,10], as confirmed by the activation energy *E*_A_ ≈ 0.55 eV of the thermally activated process. In the case of the long filling pulse, DCTs still exhibited the thermally activated response due to electron emission from Fe traps; however, an additional *T*-independent process was identified. The experimental findings were reproduced with two-dimensional device simulations including Fe traps in the buffer and a charging mechanism of the passivation/barrier interface.

The paper is organized as follows. In Section 2, we describe the devices under study (DUTs). In Section 3 we present the characterization results in terms of pulsed I-V curves and drain current transients. In Section 4 we present the simulation results. Finally, in Section 5 we draw the conclusions.

## 2. Devices under Study

**Figure 1:**
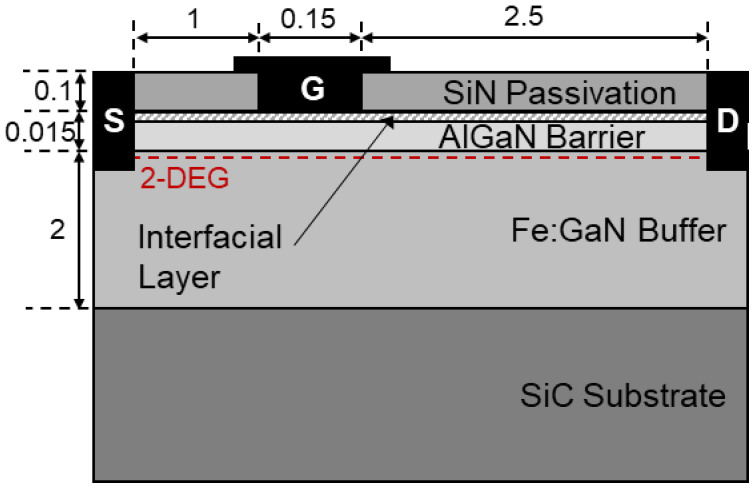
Schematic cross-section of the DUTs. 2-DEG stands for two-dimensional electron gas. A narrow (2 nm) interface layer between the passivation and AlGaN barrier was introduced in the simulations to reproduce experimental results, as discussed in more detail in Section 4. Reported dimensions are in μm (not to scale).

The devices under study (DUTs) were AlGaN/GaN HEMTs grown on SiC substrates. The AlGaN barrier layer is 15 nm thick with a 22% aluminum concentration. Iron (Fe) impurities are introduced in the 2 μm-thick GaN buffer to reduce drain-source leakage and increase breakdown voltage. Iron concentration in the GaN buffer starting from the SiC substrate interface was constant at 1 × 10^18^ cm^−3^ until 0.6 μm below the AlGaN/GaN interface, above which it decayed with a slope of one decade every 0.4 μm. The devices were passivated by a 100 nm-thick SiN layer. Gate length (*L*_G_) was 0.15 μm and gate width (*W*_G_) was 2 × 25 μm. Threshold voltage (*V*_TH_) was −2.2 V. The cross-section of the DUTs is shown in Figure 1. 

## 3. Experimental Results

Measurements were carried out by means of: (i) a custom-made drain switch that allows applying two selectable voltage levels to the drain terminal; (ii) a Keysight 33520B dual-channel waveform generator for the gate signal and drain switch control; (iii) a Keysight E3647A dual-channel power supply providing the two drain voltage levels applied during the measurements; (iv) a PicoScope 5444D four-channel 200 MHz digital sampling oscilloscope for acquiring the signals needed for *V*_D_ and *I*_D_ measurement.

### 3.1. Pulsed I-V Curves

**Figure 2:**
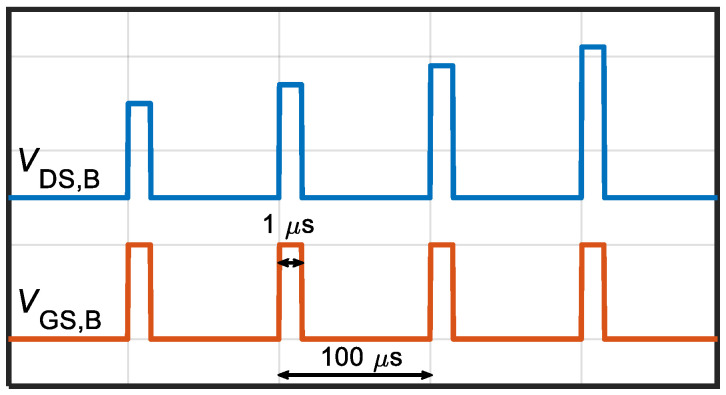
Illustration of signal waveforms employed during the pulsed I-V characterization. Short (1-μs) pulses were synchronously applied at the drain and gate terminals from the respective reference baseline (*V*_DS,B_ and *V*_GS,B_) with duty cycle 1%. The I-V curves were acquired from four different baselines, see Figure 3.

Pulsed I-V measurements were carried out by applying synchronous voltage pulses to the gate and drain terminals from a reference baseline while keeping the source contact grounded. Voltage pulses were 1 μs long with duty cycle of 1%. An illustration of the signal waveforms used for the pulsed I-V curves acquisition is shown in Figure 2

**Figure 3:**
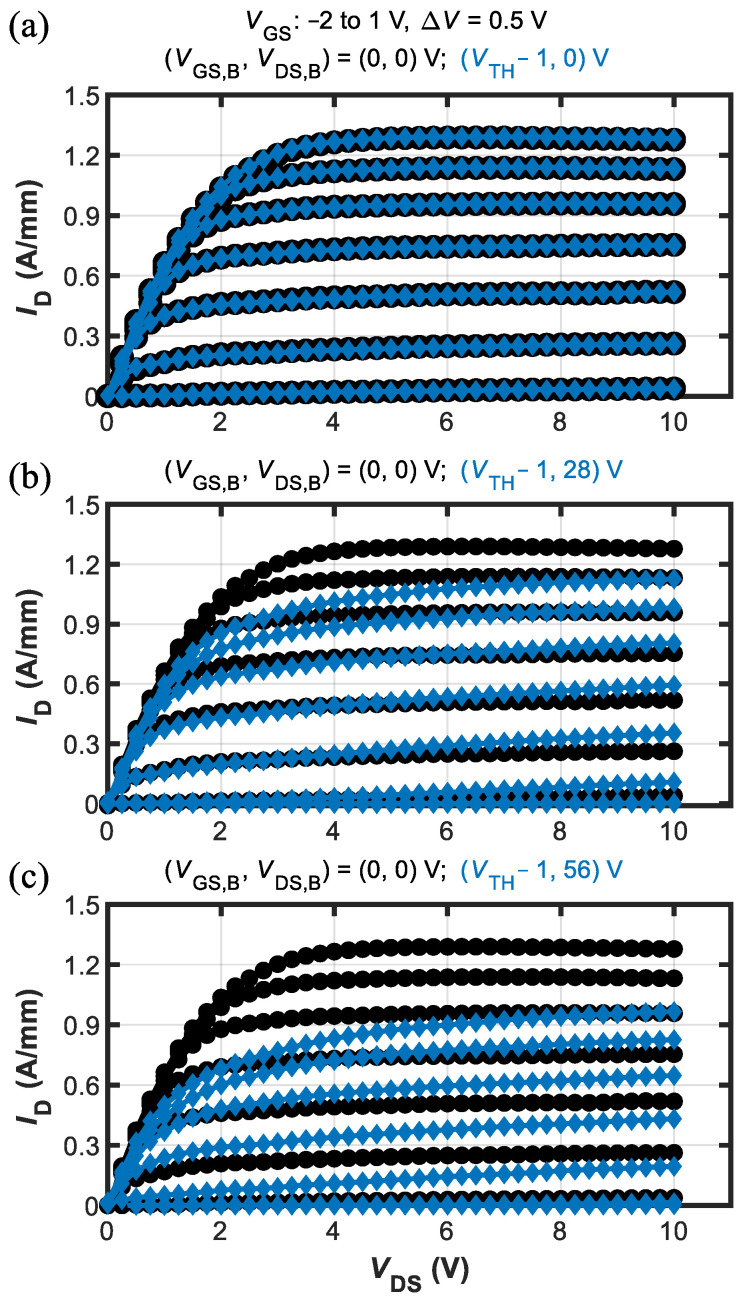
Pulsed *I*_D_-*V*_DS_ characteristics obtained from different baselines for *V*_GS_ = −2 to 1 V (Δ*V* = 0.5 V). Negligible gate-lag (assessed by pulsing from *V*_GS,B_ = *V*_TH_–1 V) is shown in panel (**a**); significant dispersion shown in panels (**b**,**c**) (assessed when pulsing from *V*_GS,B_ = *V*_TH_–1 V and *V*_DS_ = 28 or 56 V) suggests current collapse was mainly induced by deep trap states in the buffer.

Results of this kind of characterization are shown in Figure 3 in terms of output *I*_D_-*V*_DS_ curves. The output characteristics were acquired from four different baselines, namely: (*V*_GS,B_,*V*_DS,B_) = (0, 0), (*V*_TH_–1, 0), (*V*_TH_–1, 28), and (*V*_TH_–1, 56) V. Figure 3a shows the comparison between the (0,0) and (*V*_TH_–1, 0) baselines, allowing us to assess the gate-lag effect. Interestingly, in these devices no appreciable gate-lag was detected, indicating excellent stability of the device region under the gate. Figure 3b,c show the comparison between the (0,0) baseline and the (*V*_TH_–1, 28) and (*V*_TH_–1, 56), respectively. Both figures showed moderate increase of knee voltage but significant saturated current compression, especially for *I*_D_-*V*_DS_ curves at high *V*_GS_. These results indicate that trapping effects leading to current collapse were most likely happening in the gate-drain access region [2]. As such, the amount of degradation as well as its dependence on the applied *V*_DS_ were influenced by the gate-to-drain spacing.

### 3.2. Drain Current Transients

**Figure 4:**
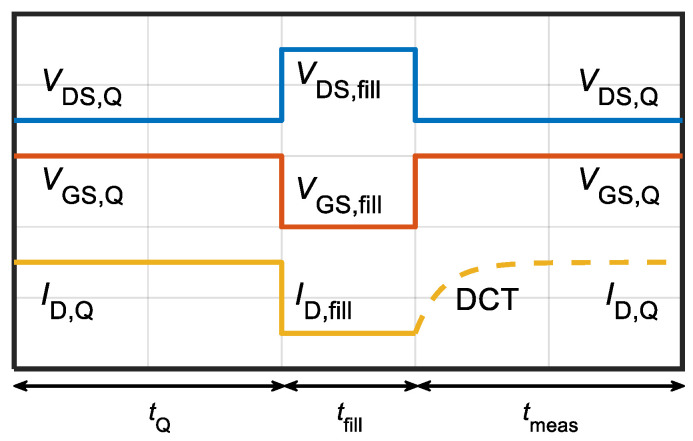
Illustration of signal waveforms employed during the DCT characterization. A short (1 μs) or long (100 s) trap-filling pulse was applied by driving the device in the OFF-state. Then, DUT was biased back at (*V*_GS,Q_, *V*_DS,Q_) and *I*_D_ was measured over several time decades up to 100 s to acquire the actual DCT. Waveform values and time intervals are not in scale.

As discussed in Section 2, the GaN HEMTs under study were intentionally Fe-doped to reduce leakage and, consequently, increase breakdown voltage. However, Fe-related traps in the buffer are well known to cause dispersion effects [9] similar to those observed in Figure 3. Therefore, the subsequent characterization by means of DCTs measurement was performed to clarify the role of Fe-related buffer traps. DCTs were acquired at several temperatures, i.e., *T* = 30–70 °C with 10 °C steps, to characterize the activation energy of the drain current recovery process. An illustration of the signal waveforms used for the DCTs acquisition is shown in Figure 4. 

**Figure 5:**
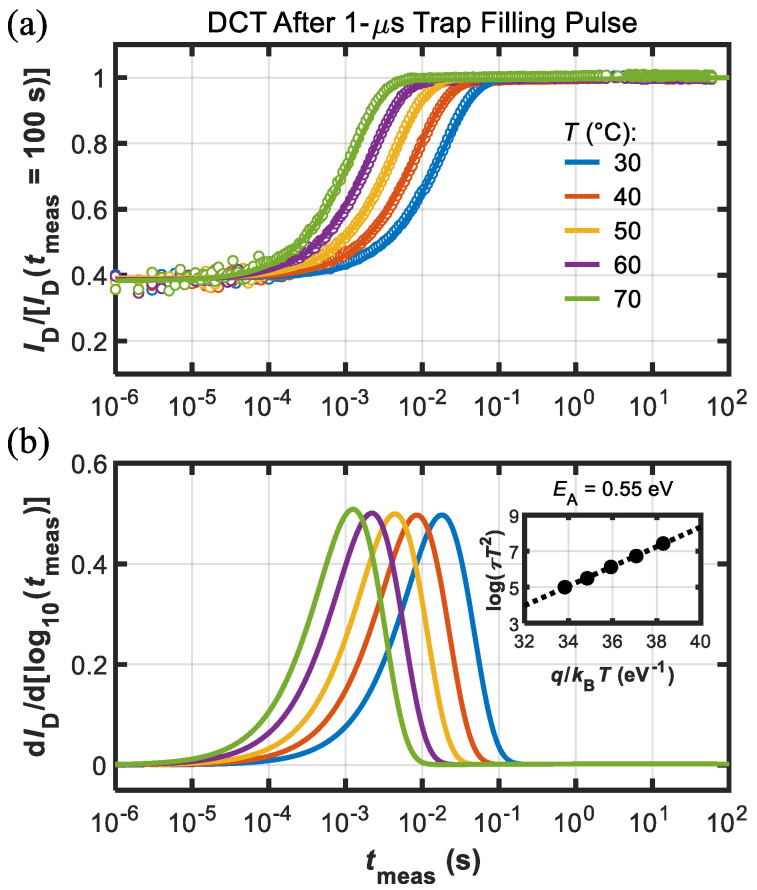
(**a**) DCTs measured at different temperature (see legend) after the short 1 us trap-filling pulse (dots are data and lines are fitting functions). Current was normalized to final value, i.e., *I*_D_(*t*_meas_ = 100 s). (**b**) d*I*_D_/d[log10(*t*_meas_)] used for the extraction of the time constants (*τ*) to build the Arrhenius plot (see inset) that yielded *E*_A_ = 0.55 eV.

The quiescent bias conditions were (*V*_GS,Q_,*V*_DS,Q_) = (−1, 2) V; during the trap-filling pulse bias was set to (*V*_GS,fill_, *V*_DS,fill_) = (*V*_TH_–1, 56) V to have a sufficient signal amplitude during DCT monitoring. Trap-filling pulse duration (*t*_fill_) was set to 1 μs, which ensured trapping in Fe-associated states [9,10]. Figure 5 shows the results of DCT monitoring after application of the short filling pulse. 

It can be observed that in Figure 5 and subsequent ones *I*_D_ during DCT was normalized to final value for the sake of comparison. Clear single-exponential waveforms can be identified in Figure 5a, which is typical of pure electron emission from point defects associated with Fe-doping [9,10,11]. To extract the activation energy (*E*_A_) of the thermally activated emission process, time constants of the DCTs at each temperature were extracted by fitting the transients with streched exponential functions [12], and determined as the point where the d*I*_D_/d[log_10_(*t*_meas_)] signals peaked (see Figure 5b). Linear fitting of the Arrhenius plot in the inset of Figure 5b allowed extracting *E*_A_ ≈ 0.55 eV, which is typical of Fe-associated traps [2,9,12]. 

**Figure 6:**
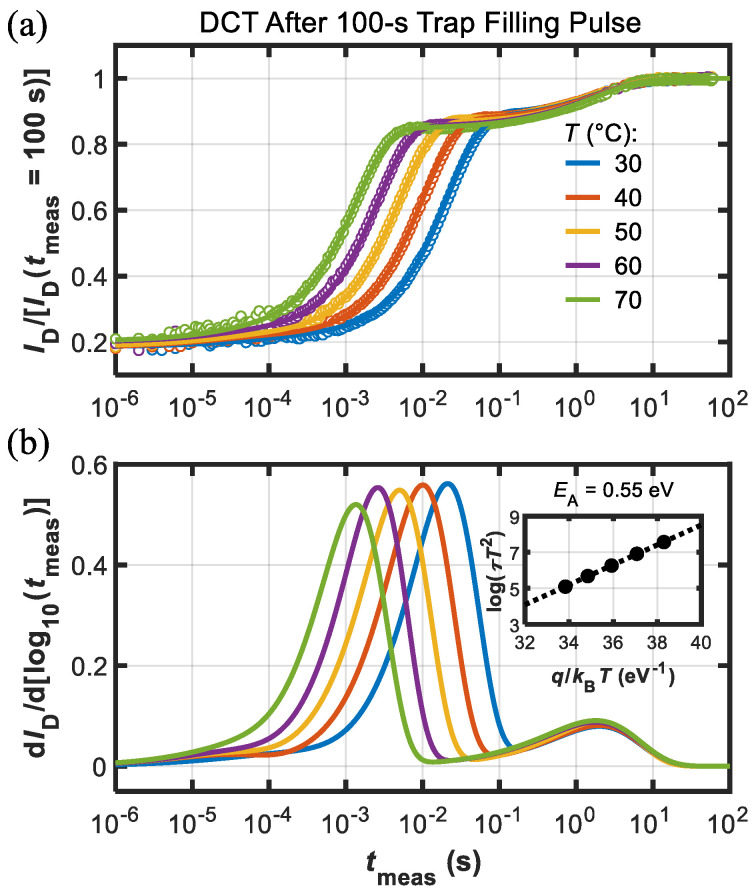
(**a**) DCTs measured at different temperatures (see legend) after the long 100 s trap-filling pulse (dots are data and lines are fitting functions). Current was normalized to final value, i.e., *I*_D_(*t*_meas_ = 100 s). (**b**) d*I*_D_/d[log10(*t*_meas_)] used for the extraction of the time constants (*τ*) to build the Arrhenius plot (see inset) that yielded *E*_A_ = 0.55 eV. In this case, another *T*-independent process was observed.

Interestingly, when applying a long trap-filling pulse (*t*_fill_ = 100 s) the DCT measurements revealed a picture richer in texture compared to the previous case. As shown by Figure 6, two separate processes could be identified. The first is analougous to the one revealed by Figure 5, and can be attributed to Fe-related traps, as confirmed by the Arrhenius plot in the inset of Figure 6b. The second process instead was characterized by time constants of about 1–2 s and negligible acceleration with temperature, as shown in Figure 6.

## 4. Simulation Results

To confirm the role of Fe-related traps shown in Figure 5 and Figure 6 and to gain more insights into the nature of the second, T-independent process identified earlier, we performed two-dimensional device simulations with the TCAD commercial software SDevice^TM^ [13]. Charge transport was modeled by means of the drift-diffusion equations. Electron mobility in the 2-DEG was set to 1800 cm^2^/V.s at room temperature [14]. Electron saturation velocity in GaN was set to 1.5 × 10^7^ cm/s. The piezoelectric polarization charge at the AlGaN/GaN interface was modeled with the default strain model of the simulator. Gate leakage current present in the Schottky junction between the metal and the AlGaN barrier was modeled by the thermionic- and field-emission mechanisms. A nonlocal tunneling model based on the WKB approximation allowed computing self-consistently the field-dependent component of the gate current [15]. Fe traps were introduced as acceptor-like states in the buffer 0.55 eV below GaN conduction band, with a capture cross-section for electrons of 1.7 × 10^−15^ cm^2^. Analogously to the actual samples, Fe trap concentration was uniform and equal to 1 × 10^18^ cm^−3^ up to 0.6 μm below the AlGaN/GaN interface, from which concentration exponentially decayed one decade per 0.4 μm [10,16]. Interestingly, the temperature-independent process put experimentally into evidence with long filling pulses could be emulated by simulations by assuming that this phenomenon was induced by electron injection from the gate into a thin (2 nm) interface layer between the passivation and the AlGaN barrier, see Figure 1. 

**Figure 7:**
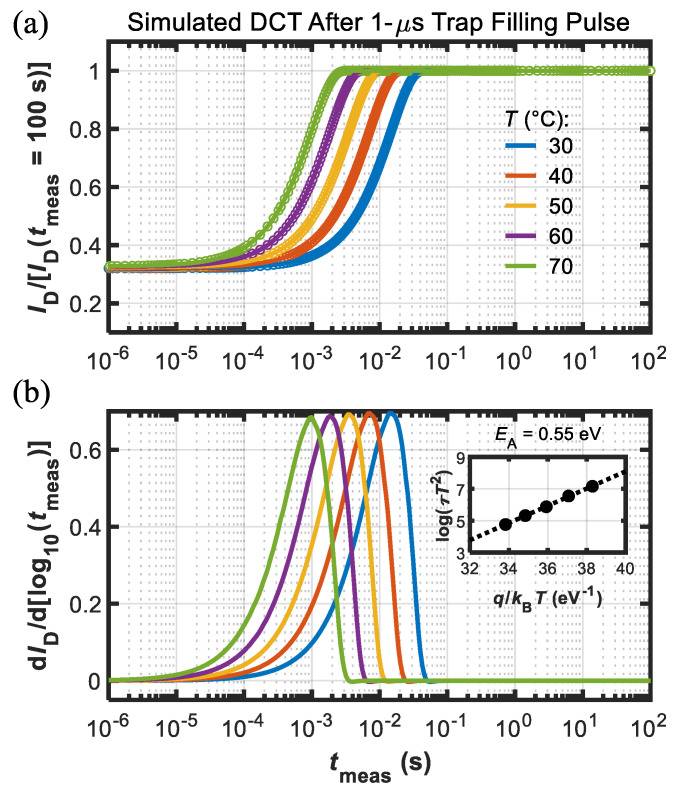
(**a**) Simulated DCTs at different temperature (see legend) after the short 1 us trap-filling pulse (dots are data and lines are fitting functions). Current was normalized to final value, i.e., *I*_D_(*t*_meas_ = 100 s). (**b**) d*I*_D_/d[log10(*t*_meas_)] used for the extraction of the time constants (*τ*) to build the Arrhenius plot (see inset) that yielded *E*_A_ = 0.55 eV.

**Figure 8:**
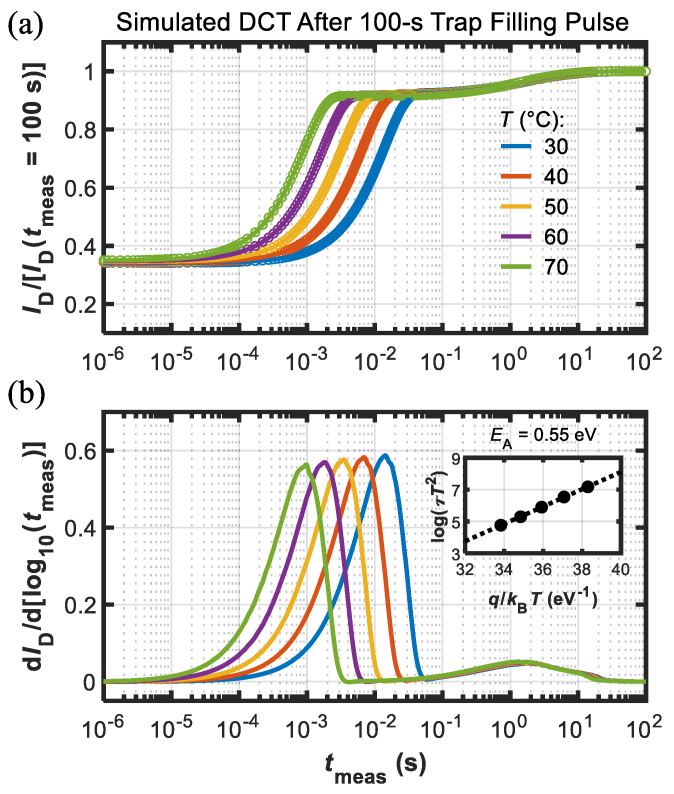
(**a**) Simulated DCTs at different temperatures (see legend) after the long 100 s trap-filling pulse (dots are data and lines are fitting functions). Current was normalized to final value, i.e., *I*_D_(*t*_meas_ = 100 s). (**b**) d*I*_D_/d[log10(*t*_meas_)] used for the extraction of the time constants (*τ*) used to build the Arrhenius plot (see inset) that yielded *E*_A_ = 0.55 eV. Both processes found in Figure 4 were reproduced by the simulations.

The electron transport through this layer, possibly governed by a hopping mechanism, is mimicked, introducing a large density of shallow levels with a very small electron capture cross-section. Simulation results in terms of DCTs, carried out under the same conditions employed during the experiments on the DUTs, are shown in Figure 7 and Figure 8 for the short (1 μs) and long (100 s) trap-filling pulses, respectively. The good agreement between simulations and experimental data indicates the soundness of the modeling framework adopted here, validating the role of Fe traps in the observed current-collapse effects and suggesting the route for a full understanding of the slow recovery process.

## 5. Conclusions

We investigated the current dispersion in short gate-length AlGaN/GaN HEMTs for 5G applications. Pulsed I-V characterization revealed the absence of pure gate-lag effects but significant current-collapse effects under double gate and drain voltage pulsing. Drain current transients (DCTs) carried out after a short 1 μs trap-filling pulse revealed that current collapse was mainly due to Fe-doping related traps in the GaN buffer. In addition, DCTs carried out after long 100 s trap-filling pulse disclosed an additional slow *T*-independent process that contributed to the total current dispersion. Two-dimensional device simulations validated the role of Fe traps and suggested that the slow process can be related to surface charging effects induced by gate electron injection and transport through the passivation/barrier interface.

## Data Availability

The data presented in this study are available on request from the corresponding author.
